# Retinal microglia express more MHC class I and promote greater T-cell-driven inflammation than brain microglia

**DOI:** 10.3389/fimmu.2024.1399989

**Published:** 2024-05-10

**Authors:** Christina L. Bloomfield, Joyce Gong, Steven Droho, Hadijat M. Makinde, Miranda G. Gurra, Cecilia H. Stumpf, Arjun Kharel, Gaurav Gadhvi, Deborah R. Winter, Weiguo Cui, Carla M. Cuda, Jeremy A. Lavine

**Affiliations:** ^1^ Department of Medicine, University of Illinois College of Medicine, Rockford, IL, United States; ^2^ Department of Ophthalmology, Feinberg School of Medicine, Northwestern University, Chicago, IL, United States; ^3^ Division of Rheumatology, Department of Medicine, Feinberg School of Medicine, Northwestern University, Chicago, IL, United States; ^4^ Division of Biostatistics, Department of Preventive Medicine, Feinberg School of Medicine, Northwestern University, Chicago, IL, United States; ^5^ Department of Pathology, Feinberg School of Medicine, Northwestern University, Chicago, IL, United States

**Keywords:** antigen presentation, microenvironment, microglia, MHC class I, retina, brain

## Abstract

**Introduction:**

Macrophage function is determined by microenvironment and origin. Brain and retinal microglia are both derived from yolk sac progenitors, yet their microenvironments differ. Utilizing single-cell RNA sequencing (scRNA-seq) data from mice, we tested the hypothesis that retinal and brain microglia exhibit distinct transcriptional profiles due to their unique microenvironments.

**Methods:**

Eyes and brains from 2-4 month wildtype mice were combined (20 eyes; 3 brains) to yield one biologically diverse sample per organ. Each tissue was digested into single cell suspensions, enriched for immune cells, and sorted for scRNA-seq. Analysis was performed in Seurat v3 including clustering, integration, and differential expression. Multi-parameter flow cytometry was used for validation of scRNA-seq results. Lymphocytic choriomeningitis virus (LCMV) Clone 13, which produces a systemic, chronic, and neurotropic infection, was used to validate scRNA-seq and flow cytometry results *in vivo*.

**Results:**

Cluster analysis of integrated gene expression data from eye and brain identified 6 *Tmem119* ^+^ *P2ry12* ^+^ microglial clusters. Differential expression analysis revealed that eye microglia were enriched for more pro-inflammatory processes including antigen processing via MHC class I (14.0-fold, *H2-D1* and *H2-K1*) and positive regulation of T-cell immunity (8.4-fold) compared to brain microglia. Multi-parameter flow cytometry confirmed that retinal microglia expressed 3.2-fold greater H2-Db and 263.3-fold more H2-Kb than brain microglia. On Day 13 and 29 after LCMV infection, CD8^+^ T-cell density was greater in the retina than the brain.

**Discussion:**

Our data demonstrate that the microenvironment of retina and brain differs, resulting in microglia-specific gene expression changes. Specifically, retinal microglia express greater MHC class I by scRNA-seq and multi-parameter flow cytometry, resulting in a possibly enhanced capability to stimulate CD8^+^ T-cell inflammation during LCMV infection. These results may explain tissue-specific differences between retina and brain during systemic viral infections and CD8^+^ T-cell driven autoimmune disease.

## 1 Introduction

Macrophages are heterogeneous innate immune cells whose function is dependent upon their origin and microenvironment. Microglia are a specialized subtype of macrophage, resident in the brain and neural retina. Both retinal and brain microglia originate from the same primitive erythro-myeloid precursor in the yolk sac and migrate to their respective tissues during early embryonic development, where they self-renew and reside for life (1, 2). Additionally, both retinal and brain microglia express specialized markers such as *Tmem119* (3, 4) and *P2ry12* (5, 6). However, the brain and retinal microenvironments are not identical, including a lack of myelination in the retina and the retina’s close proximity to the choroid, which is not protected by the blood-ocular barrier. This is in contrast to the brain, wherein myelination is extensive and the majority of the brain is not within 200–300 µm of the leptomeninges or choroid plexus.

The microenvironment is also an important determinant of macrophage function (7). In the brain, several specialized populations of macrophages have been described at distinct CNS borders, including dural, leptomeningeal, perivascular, and choroid plexus macrophages (8). While dural, leptomeningeal, and perivascular macrophages are long-lived, choroid plexus macrophages are derived from blood monocytes (8). Additionally, dural and choroidal plexus macrophages highly express MHC class II genes, while leptomeningeal macrophages are enriched for markers like *Lyve1*, *Folr2*, and *Pr2rx7* (9), suggesting that ontogeny alone does not dictate gene expression or function. Similarly, in the eye, using both multiparameter flow cytometry and fate mapping studies, three distinct nonmicroglial macrophage subsets were identified, each of distinct origin and MHCII expression (10). Thus, the distinct retinal and cerebral microenvironments likely drive differences in microglia function between organs.

There are prior reports that suggest both similarities and differences between retinal and brain microglia activation and function. The disease-associated microglia (DAM) phenotype is a specific transcriptional signature first found in a subtype of brain microglia that correlates with Alzheimer’s disease (AD) in mice and humans (11). This transcriptional signature was additionally found in neuro-protective retinal microglia in mouse models of neurodegeneration (1). Although still debated, the DAM phenotype is believed to be similarly neuroprotective as opposed to the disease-inflammatory macrophages that are believed to be degenerative (12). Perhaps most intriguing, differences between retinal and brain microglia are suggested by the opposing genetic risk in diseases such as AD and age-related macular degeneration (AMD). The apolipoprotein E4 allele (ApoE4) is a recognized risk factor for AD (13), but it appears to have a protective role in AMD (14). Conversely, the ApoE2 allele, a known risk factor for AMD, has been associated with a reduced risk of AD (15, 16). Microglia are known to express ApoE, and alterations in microglia function lead to exacerbated AMD phenotypes (17). Thus, in two microglia-driven diseases, altered ApoE function can have opposing effects on microglia function and disease progression, suggesting tissue microenvironment-driven responses.

In this study, we compared gene expression between retinal and brain microglia using single-cell RNA-sequencing (scRNA-seq) data to infer how microenvironment impacts function. We found that retinal microglia are generally more proinflammatory and express higher levels of MHC class I and class II genes. We further validated by multiparameter flow cytometry that retinal microglia present with increased surface expression of MHC class I than brain microglia. Systemic infection with lymphocytic choriomeningitis virus (LCMV) clone 13, which produces a systemic, chronic, and neurotropic infection, was used to validate scRNA-seq and flow cytometry results *in vivo*. We found that T-cell density was significantly greater in the retina than the brain after LCMV infection. Our findings may explain tissue-specific differences between the retina and brain during systemic viral infections and T-cell-driven autoimmune diseases.

## 2 Results

To gain insight into the functional impact of the tissue-specific microenvironment of microglia, we performed scRNA-seq of immune cell isolates from brain and eye tissues. Brains from three wild-type female mice aged 10–12 weeks were harvested and dissected to remove meninges. Eyes from 10 wild-type female mice aged 10–12 weeks were enucleated and dissected to remove conjunctiva, orbital fat, optic nerve, and extraocular muscles. Each tissue was individually combined to yield a biologically diverse sample per organ. Brains were digested into a single cell suspension and stained for viability dye, CD45, lineage markers (CD4, CD8, B220, NK1.1, Ly6G, SiglecF), and CD11b (**Table 1**) to sort live CD45^+^CD11b^+^Lin^neg^ mononuclear phagocytes for scRNA-seq. Eyes were digested into a single-cell suspension and stained for viability dye, CD45, and CD11b (**Table 1**) to sort CD45^+^ cells for scRNA-seq to increase yield due to the smaller tissue compared to brains.

**Table 1:** Antibodies used for flow cytometry and immunofluorescence.

Antibody	Fluorophore	Manufacturer, product No., dilution	Usage
Rat anti-mouse CD16/CD32	–	BD Biosciences, 553142	*F_c_* block
Aqua Live/Dead	AmCyan	Thermo Fisher Scientific, 65-0866-14	All flow cytometry
Rat anti-mouse CD45	BUV396	BD Biosciences, 564279	Flow cytometry analysis
Mouse anti-mouse CD64	BV786	BD Biosciences, 741024	Flow cytometry analysis
Rat anti-mouse CD11b	APC-Cy7	BD Biosciences, 557657	Flow cytometry analysis
Rat anti-mouse B220	PE-CF594	BD Biosciences, 562313	Flow cytometry analysis
Rat anti-mouse CD4	PE-CF594	BD Biosciences, 562314	Flow cytometry analysis
Rat anti-mouse CD8	PE-CF594	BD Biosciences, 562315	Flow cytometry analysis
Rat anti-mouse Ly6G	PE-CF594	BD Biosciences, 562700	Flow cytometry analysis
Mouse anti-mouse NK 1.1	PE-CF594	BD Biosciences, 562864	Flow cytometry analysis
Rat anti-mouse Siglec-F	PE-CF594	BD Biosciences, 562757	Flow cytometry analysis
Mouse anti-mouse Cx3cr1	BV650	BioLegend, 149033	Flow cytometry analysis
Mouse anti-mouse H2-Kb	Alexa Fluor 700	Thermo Fisher Scientific, 56-5958-82	Flow cytometry analysis
Mouse anti-mouse H2-Db	PE-Cy7	Thermo Fisher Scientific, 25-5999-82	Flow cytometry analysis
Rat anti-mouse MHC II	PE	Thermo Fisher Scientific, 12-5321-82	Flow cytometry analysis
Hamster anti-mouse CD11c	BV421	BD Biosciences, 562782	Flow cytometry analysis
Rat anti-mouse CD19	PE	BD Biosciences, 553786	Flow cytometry compensation
Rat anti-mouse CD19	Alexa Fluor 700	BD Biosciences, 557958	Flow cytometry compensation
Rat anti-mouse CD8	–	Invitrogen, 14-0081-85, 1:250	Primary (IF)
Rat anti-mouse LCMV	–	Bio Cell, BE0106, 1:1000	Primary (IF)
Rabbit anti-mouse IBA-1	–	WAKO, 019-19741, 1:500	Primary (IF)
Goat anti-mouse CD31	–	R&D Systems, AF3628, 1:250	Primary (IF)
Donkey anti-rat	Alexa Fluor 555	Thermo Fisher Scientific, A78945, 1:500	Secondary (IF)
Donkey anti-rabbit	Alexa Fluor 647	Thermo Fisher Scientific, A31573, 1:500	Secondary (IF)
Donkey anti-goat	Alexa Fluor 488	Thermo Fisher Scientific, A48259, 1:500	Secondary (IF)

**Figure 1:**
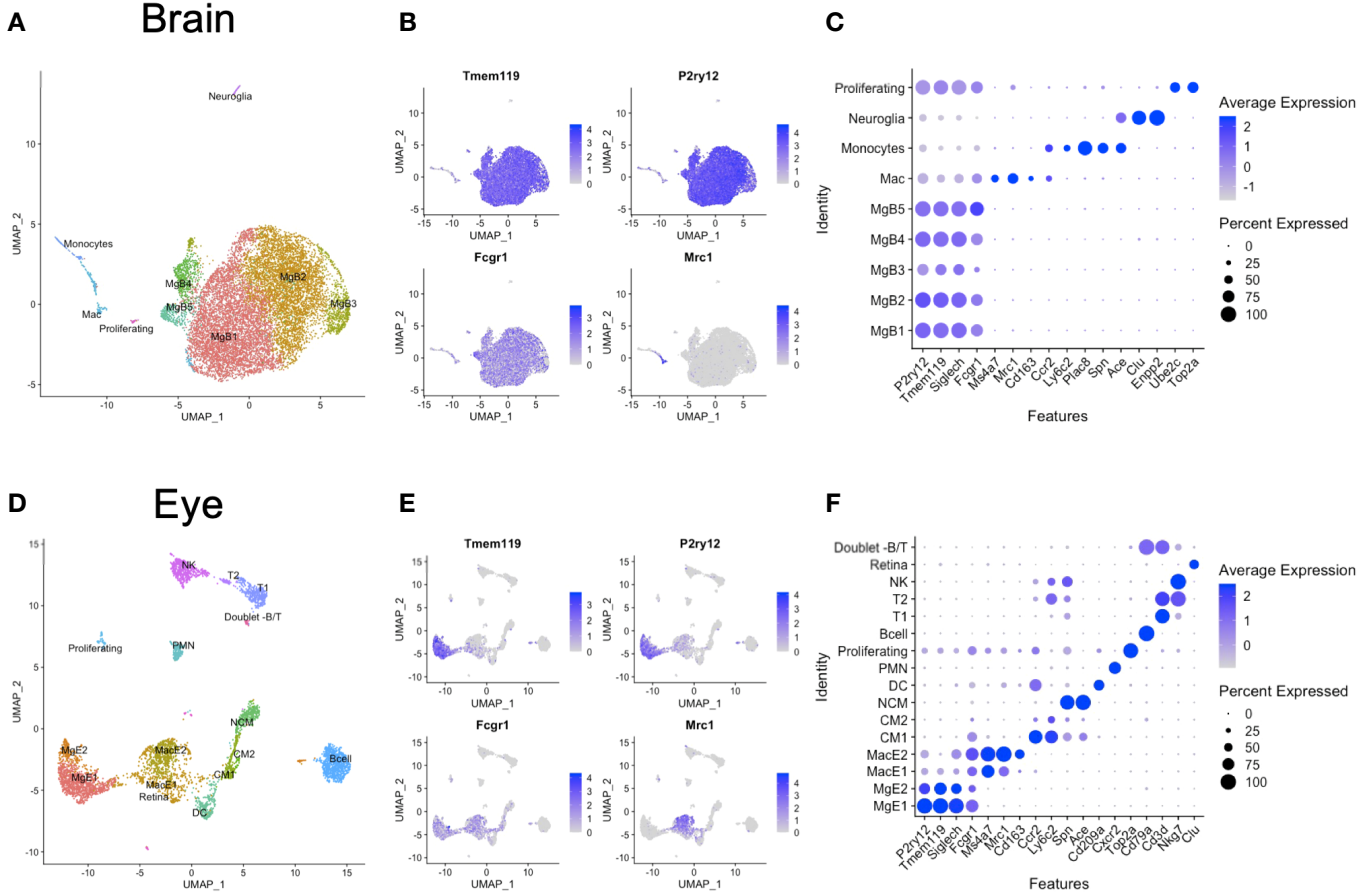
Unintegrated scRNA-seq analysis of brain and eye CD45^+^ immune cells. **(A)** Dimensionality reduction with uniform manifold approximation and projection (UMAP) for the brain. **(B)** Canonical marker genes are displayed for brain microglia and macrophage clusters in feature plots. **(C)** Canonical marker genes are displayed for each cellular cluster in the brain in a dot plot. **(D)** Dimensionality reduction with UMAP for the eye. **(E)** Canonical marker genes are displayed for eye microglia and macrophage clusters in feature plots. **(F)** Canonical marker genes are displayed for each cellular cluster in the eye in a dot plot. Mg, microglia; Mac, macrophage; CM, classical monocyte; NCM, nonclassical monocyte; DC, dendritic cell; PMN, polymorphonuclear neutrophil; NK cell, natural killer cell.

Brain and eye data were initially analyzed individually to identify the microglia and macrophages among other immune cell clusters. From the brain, we identified microglia 1–5 (MgB1-5) as *P2ry12^+^Tmem119^+^Siglech^+^Fcgr1^+^* cells and one macrophage cluster as uniquely *P2ry12* ^neg^ *Tmem119* ^neg^ *Mrc1* ^+^ *Fcgr1^+^* (Mac) (**Figures 1A–C**). In addition, we found *Ccr2^+^Ly6c2^+^Plac8^+^Spn^+^Ace^+^* monocytes and rare clusters of *Clu^+^Enpp2^+^* neuroglia and *Ube2c^+^Top2a^+^* proliferating cells (**Figures 1A–C**). From the eyes, we identified two microglia (MgE1-2) and two macrophage clusters (MacE1-2) (**Figures 1D–F**). Eye microglia were *P2ry12^+^Tmem119^+^Fcgr1^+^* cells, and macrophages uniquely *P2ry12^neg^Tmem119^neg^Fcgr1^+^Mrc1* ^+^ (**Figures 1D–F**). Monocytes were distinguished between classical monocytes (CM1-2) as *Ccr2^+^Ly6c2^+^ cells* and nonclassical monocytes (NCM) as *Spn^+^Ace^+^* cells. We additionally delineated *Cd209a^+^* DCs, *Cxcr2^+^* polymorphonuclear cells (PMN), *Top2a^+^* proliferating cells, and *Cd79a^+^* B cells. Two *Cd3d^+^* T-cell clusters were demonstrated as *Cd3d^+^Nkg7^neg^* cells (T1) and *Cd3d^+^Nkg7^+^* cells (T2), in addition to *Cd3d^neg^Nkg7^+^* NK cells. Finally, a few contaminating retinal cells (*Clu^+^* [Muller glia]) and rare B- and T-cell doublets were also identified (**Figure 1D**).

**Figure 2:**
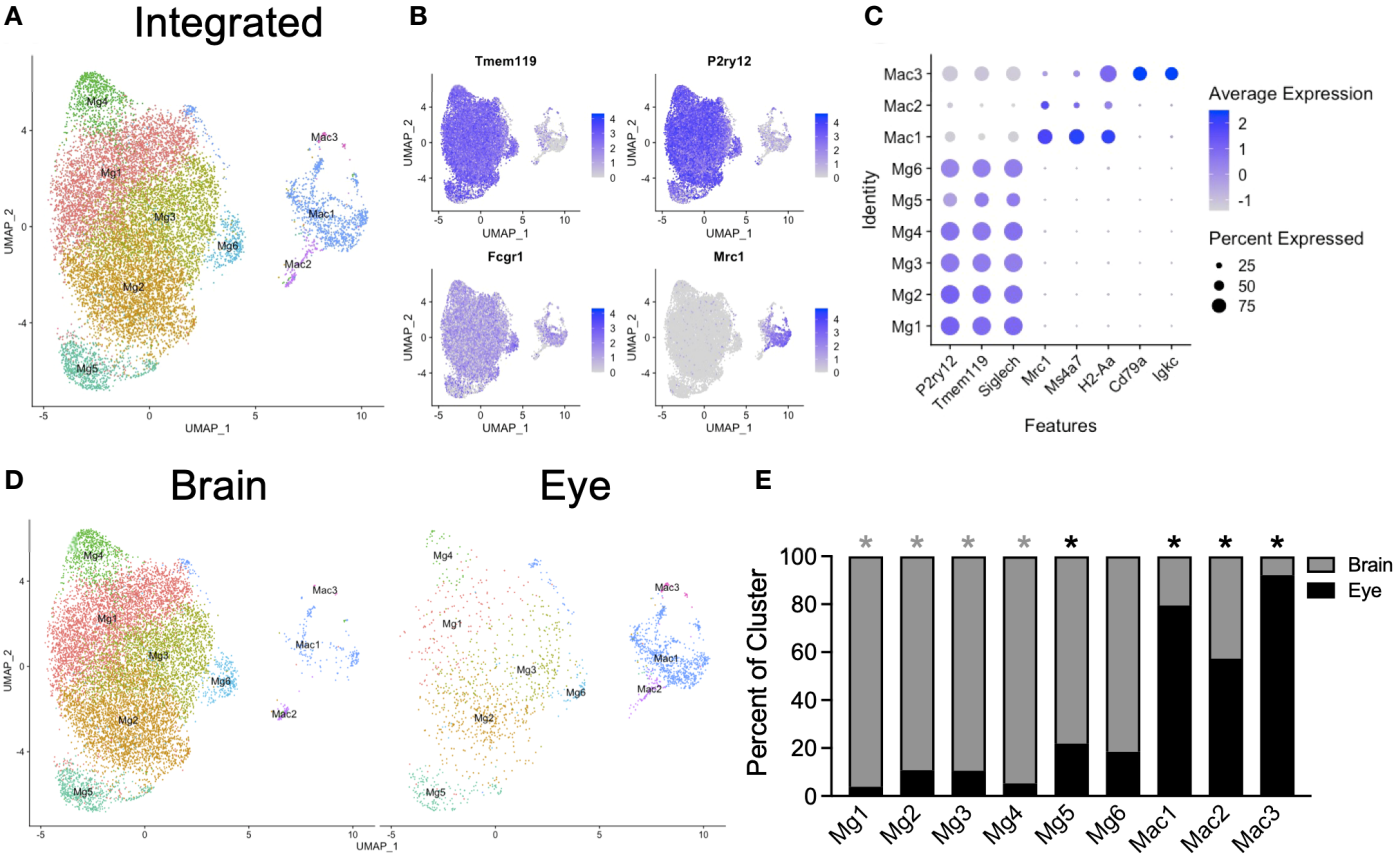
Integrated scRNA-seq analysis of brain and eye macrophages. **(A)** Dimensionality reduction with UMAP for integrated macrophage data. **(B)** Canonical marker genes are displayed for brain microglia and macrophage clusters in feature plots. **(C)** Canonical marker genes are displayed for each cellular cluster in a dot plot. **(D)** The contribution of the experimental tissue to each cluster is visualized in the faceted UMAP plot. **(E)** The contribution of each to the two tissue clusters is visualized with a stacked bar chart. Mg, microglia; Mac, macrophage. * p<0.05. Color of asterisk indicates eye vs brain.

To explore microglia heterogeneity across tissues, we subsetted the data into a macrophage/microglia subgroup for each tissue, including MgB1-5 and Mac from the brain and MgE1-2 and MacE1-2 from the eye. We included nonmicroglia macrophages in this step and made the distinction between macrophages and microglia in subsequent analysis to avoid loss of microglia. Each subset was next integrated and reclustered (**Figure 2A**). We identified six *P2ry12^+^Tmem119^+^Fcgr1^+^Mrc1^neg^* microglia subsets (Mg1-6) and two *P2ry12^neg^Tmem119^neg^Fcgr1^+^Mrc1* ^+^ macrophage subsets (Mac1-2, **Figures 2B, C**). Additionally, a contaminating macrophage subset with B-cell (*Cd79a*, *Igkc*) markers was found (Mac3), likely representing a minor doublet population (**Figure 2C**). Data from brain and eye samples contributed to each cluster (**Figures 2D, E**). Mg1-4 was enriched from the brain, Mg5 was enriched from the eye, and Mg6 demonstrated no significant enrichment (**Figure 2E**). Clusters Mac1-3 were all enriched from eye samples, likely due to the fact that brain samples had dural meninges removed and eye samples included macrophages from the iris, ciliary body, choroid, and cornea (**Figure 2E**). Although the eye data had greater sequencing depth, the canonical microglia marker genes *Tmem119* and *P2ry12* were highly expressed in brain and retinal microglia, with greater levels found in the brain despite lower sequencing depth (**Supplementary Figure S1**). Similarly, the number of genes detected was similar between brain and retina, with a slightly higher median number of genes detected in the eye (2,028 *vs*. 1,917, **Supplementary Figure S1**). These data demonstrate similarly high-quality microglia between samples for differential expression (DE) analysis.

**Figure 3:**
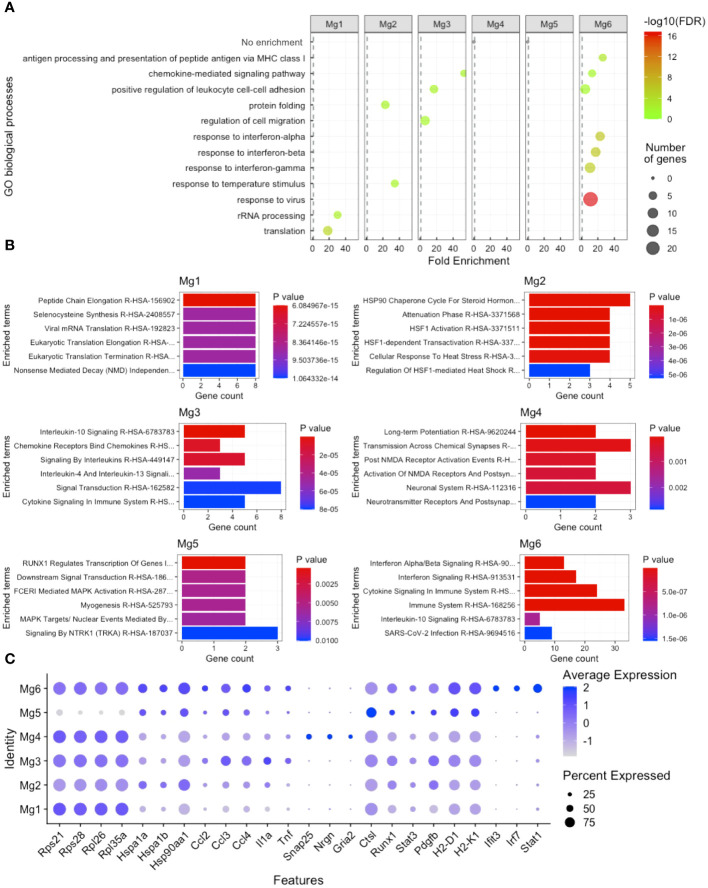
Microglia heterogeneity analysis. **(A)** Gene ontology (GO) enrichment of cluster-based DE genes. **(B)** Enrichr pathway analysis of cluster-based DE genes. **(C)** Dot plot visualization of cluster-based DE genes driving GO and pathway enrichment. FDR, false-discovery rate.

To gain insight into how microglia subpopulations differ from each other, we performed DE analysis, comparing each of the six microglia clusters with all other microglia clusters, followed by Gene Ontology (GO) and pathway analysis enrichment. We found that Mg1 was enriched for rRNA processing and translation by both the GO process (**Figure 3A**) and Enrichr pathway analysis (**Figure 3B**), including *Rps21*, *Rps28*, *Rpl26*, and *Rpl35a* (**Figure 3C**). Mg2 were enriched for protein folding and temperature response to the stimulus by both the GO process (**Figure 3A**) and pathway analysis (**Figure 3B**), including *Hspa1a*, *Hspa1b*, and *Hsp90aa1* (**Figure 3C**). Mg3 displayed a transcriptional profile enriched for chemokine-mediated signaling, regulation of cell migration, and positive regulation of leukocyte cell–cell adhesion by both the GO process (**Figure 3A**) and pathway analysis (**Figure 3B**), including *Ccl2*, *Ccl3*, *Ccl4*, *Tnf*, and *Il1a* (**Figure 3C**). Mg4-expressed synapse-regulating genes like *Snap25*, *Nrgn*, and *Gria2* (**Figure 3C**) did not demonstrate enrichment on GO analysis (**Figure 3A**) but were enriched for synapse terms by pathway analysis (**Figure 3B**). Mg5 displayed no GO enrichment and was enriched for RUNX1 transcription and MAPK targets, including DE genes *Runx1*, *Ctsl*, *Stat3*, and *Pdgfb* (**Figures 3A–C**). Finally, Mg6 was enriched for antigen processing and presentation of peptide antigen via MHC class I, chemokine-mediated signaling, positive regulation of leukocyte cell–cell adhesion, responses to the virus, interferon-alpha, interferon-beta, and interferon-gamma by both the GO process (**Figure 3A**) and pathway analysis (**Figure 3B**). Enriched genes that drove this signature included *H2-D1*, *H2-K1*, *Ifit3*, *Stat1*, and *Irf7* (**Figure 3C**). These results indicate that Mg6, which was not enriched from the eye or brain, expressed a more proinflammatory and antigen-presenting (via class I) transcriptional profile signature.

**Figure 4:**
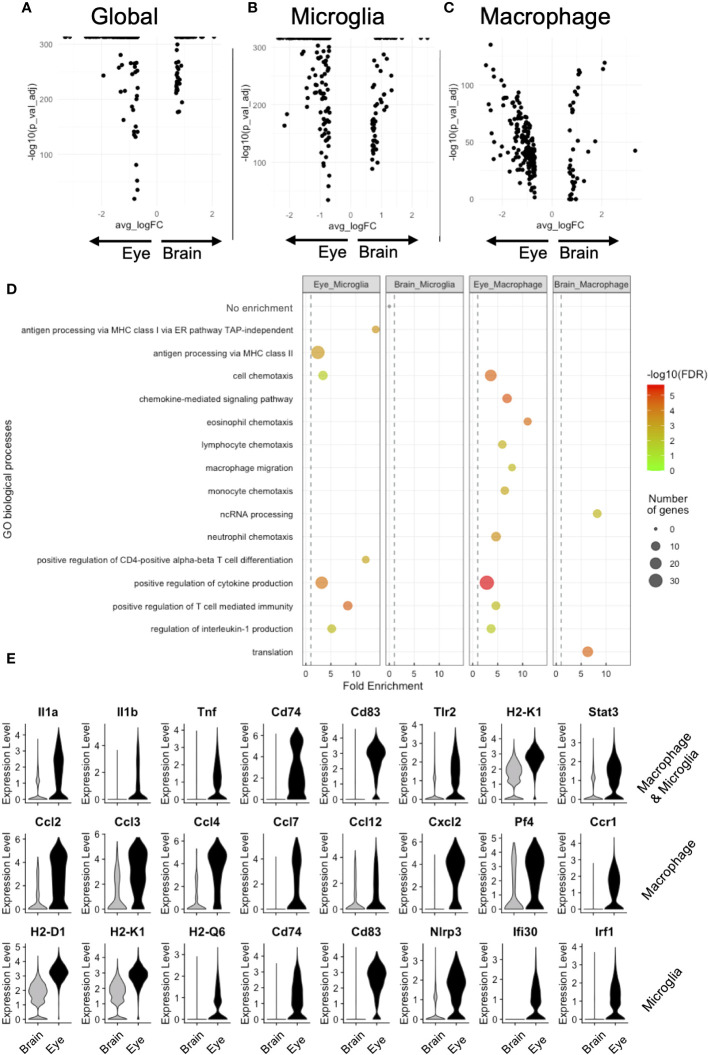
DE analysis demonstrates greater antigen presentation via MHC class I and inflammation in eye microglia compared to brain microglia. Volcano plots for global **(A)**, microglia **(B)**, and macrophage **(C)** DE genes. **(D)** GO enrichment of DE genes for eye and brain microglia subsets. **(E)** Violin plot visualization of DE genes associated with GO processes. FDR, false-discovery rate.

Next, we performed DE between brain and eye, comparing microglia and macrophages (global, **Figure 4A**), microglia (microglia, *N* = 11,633 brain and 1,165 retinal microglia, **Figure 4B**), and nonmicroglia macrophages (macrophage, *N* = 310 brain and 1,071 eye macrophages, **Figure 4C**). Global DE revealed 77 and 201 enriched genes in the brain and eye, respectively (**Figure 4A**). Microglia DE showed 56 and 170 enriched genes in the brain and retina, respectively (**Figure 4B**). Macrophage DE demonstrated 35 and 189 enriched genes in the brain and eye, respectively (**Figure 4C**). To infer functional differences between brain and eye macrophage subtypes, we performed a GO analysis of DE genes. The 56 enriched brain microglia genes showed no GO enrichment (**Figure 4D**). The 35 brain-enriched macrophage genes showed GO enrichment for translation and ncRNA processing (**Figure 4D**). Alternatively, DE enriched from ocular microglia and macrophages displayed GO enrichment for positive regulation of cytokine production (*Il1b*, *Il1a*, *Cd74*, *Cd83*, and *Tnf*), T-cell-mediated immunity (*Il1b* and *H2-K1*), and regulation of interleukin-1 production (*Tlr2*, *Tnf*, and *Stat3*, **Figures 4D, E** [top row]). DE genes enriched from ocular macrophages demonstrated GO enrichment for eosinophil, lymphocyte, monocyte, neutrophil, and macrophage chemotaxis as well as chemokine-medicated signaling pathway (genes = *Cxcl2*, *Ccl2*, *Ccl3*, *Ccl4*, and *Ccl12* and *Cxcr4*, *Ccr1*, *Ccl7*, and *Pf4*, **Figures 4D, E** [middle row]). Finally, DE genes enriched from ocular microglia showed GO enrichment for antigen processing via MHC class I (*H2-D1* and *H2-K1*) and class II (*Cd74* and *Ifi30*) and positive regulation of CD4-positive T-cell differentiation (*Cd83*, *Nlrp3*, and *Irf1*, **Figures 4D, E** [bottom row]). These data suggest that ocular microglia might be potentially better antigen presenters to T cells than brain microglia.

**Figure 5:**
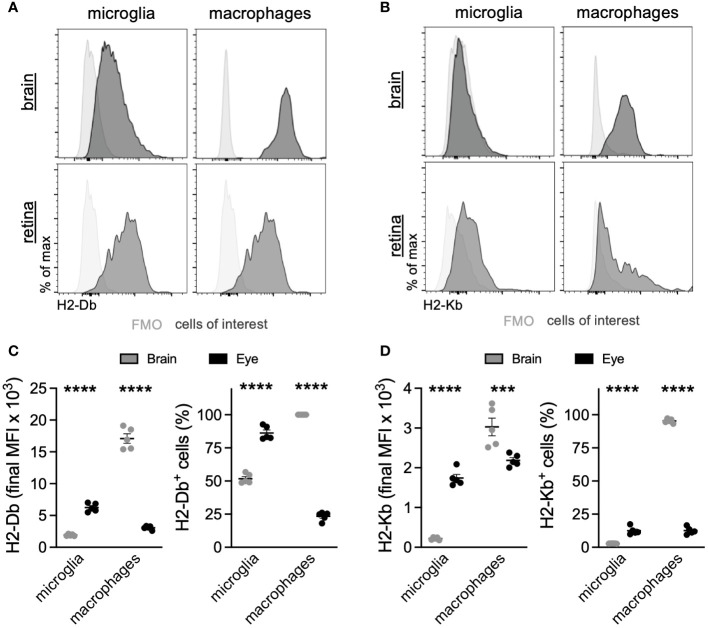
Retinal microglia express greater MHC class I than brain microglia. Representative multiparameter flow cytometry histograms of H2-Db **(A)** and H2-Kb **(B)** from the brain and retina. Retinal microglia express greater H2-Db **(C)** and H2-Kb **(D)** than brain microglia by both mean fluorescence intensity (MFI) and percent of cells using fluorescence minus one (FMO) controls to define positive staining. ^***^ *p* < 0.001; ^****^ *p* < 0.0001. N = 5 mice per group.

Based upon the microglia DE between the eye and brain, which revealed that ocular microglia may present antigen via MHC class I differently than brain microglia, we sought to validate MHC class I expression by multiparameter flow cytometry. The scRNA-seq data showed greater expression of MHC class I genes *H2-D1*, *H2-K1*, *H2-Q6*, and *H2-Q7* in retinal microglia compared to brain microglia (**Figure 4E**). These differences were also present in ocular macrophages, but to a lesser degree (**Supplementary Figures S2A, B**). From the same perfused *Tmem119^GFP/+^* mouse, brains without meninges and retinas were isolated and processed for multiparameter flow cytometry analysis. Gating strategies for the brain (**Supplementary Figure S3**) and retina (**Supplementary Figure S4**) can be found in the data supplement. We identified four populations of interest: microglia, and lymphocytes. Representative histograms for H2-Db and H2-Kb expression in each population of interest are shown in **Figures 5A, B**. Similar to gene expression data from scRNA-seq, retinal microglia demonstrated significantly greater H2-Db and H2-Kb expression by both MFI and percent-positive cells compared to brain microglia (**Figures 5C, D**). Alternatively, retinal nonmicroglial macrophages, which include perivascular macrophages and vitreal hyalocytes (18), expressed lower levels of H2-Db and H2-Kb compared to brain border-associated macrophages. These data suggest that retinal microglia express greater MHC class I (H2-Kb and H2-Db) compared to brain microglia, validating our scRNA-seq data.

**Figure 6:**
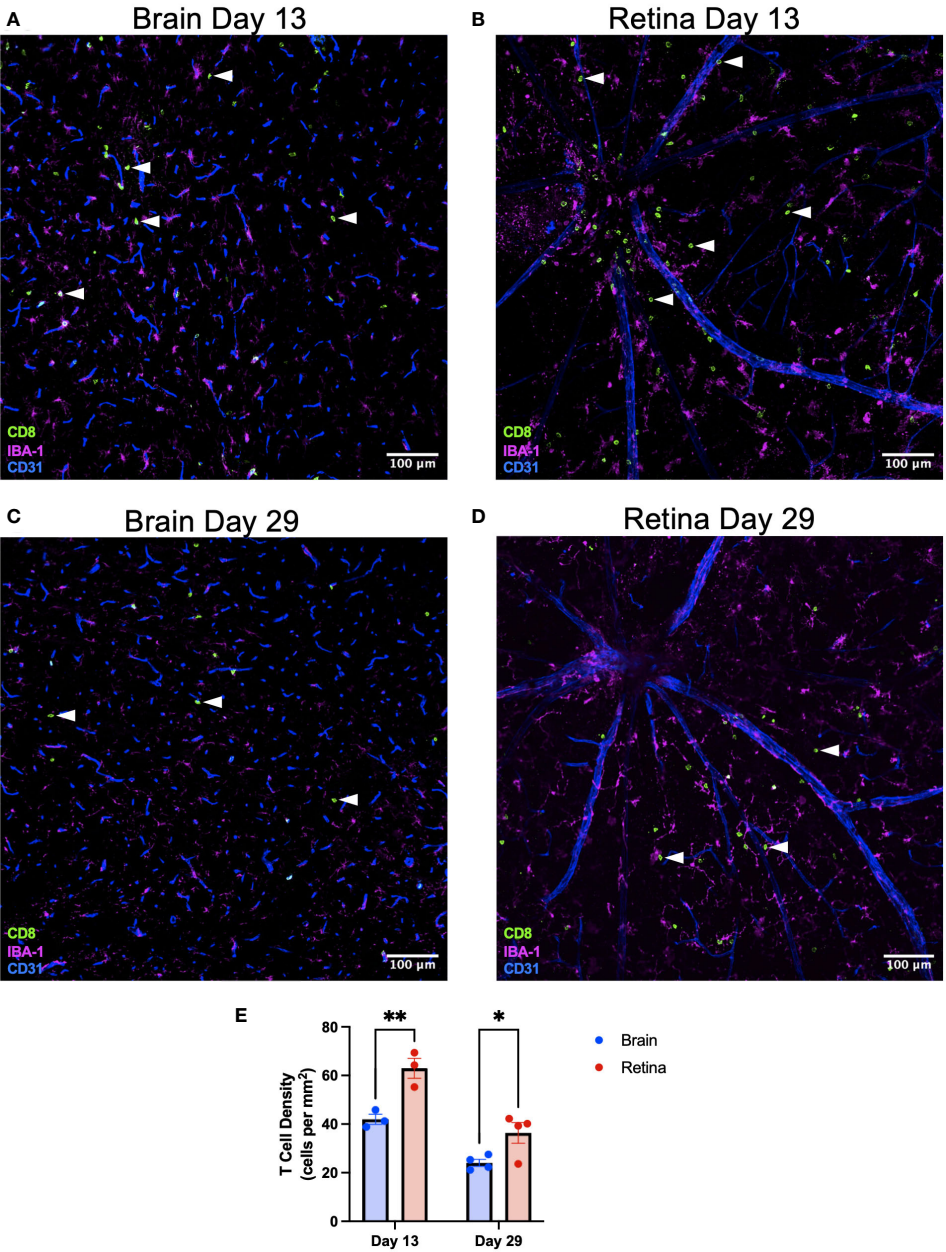
CD8^+^ T-cell density is greater in the retina than the brain after systemic LCMV clone 13 infection. Representative immunofluorescence image of a ×20 brain section identifying CD8^+^ T cells with white arrowheads on day 13 **(A)** and day 29 **(C)** postinfection. Scale bars are 100 µm. Representative immunofluorescence image of a ×20 retinal flat-mount section identifying CD8^+^ T cells with white arrowheads on day 13 **(B)** and day 29 **(D)** postinfection. **(E)** CD8^+^ T-cell density is greater in the retina than the brain on days 13 and 29. ^*^ *p* < 0.05; ^**^ *p* < 0.01. N = 3–4 mice per group.

To test the biological importance of our findings that retinal microglia express greater MHC class I and higher levels of T-cell differentiation genes, we sought to challenge the retina and brain equally with a systemic, neurotrophic viral infection that would be presented on MHC class I. Wild-type mice were infected intravenously (via retro-orbital injection) with LCMV clone 13, which causes a persistent, systemic, and neurotrophic infection (19, 20). Retina and brain were isolated at an early (day 13) and late (day 29) time point. Based upon our findings that retinal microglia express greater MHC class I and more T-cell differentiation genes, we hypothesized that CD8^+^ T-cell density would be greater in the retina than the brain. See **Supplementary Figures S5A–C** for the entire midsagittal brain sections from each animal from day 13. CD8^+^ T-cell density was heterogeneous but was not found to be region-specific. We found that CD8^+^ T-cell density was significantly greater in the retina than in the brain (**Figure 6**) on day 13 (*p* < 0.01) and day 29 (*p* < 0.05). CD8^+^ T-cell density in the retina was greater in the superficial than in deep retinal layers (**Supplementary Figures S5D–F**). This likely occurred because immune cell extravasation occurs at postcapillary venules, which exist in the superficial retina, and not in the capillaries of the deep retina (21). To confirm that the retro-orbital injection did not lead to differential responses between eyes, T-cell densities were compared between eyes on retinal flat mounts on day 15. No significant difference was detected in T-cell density between eyes (**Supplementary Figure S5**).

**Figure 7:**
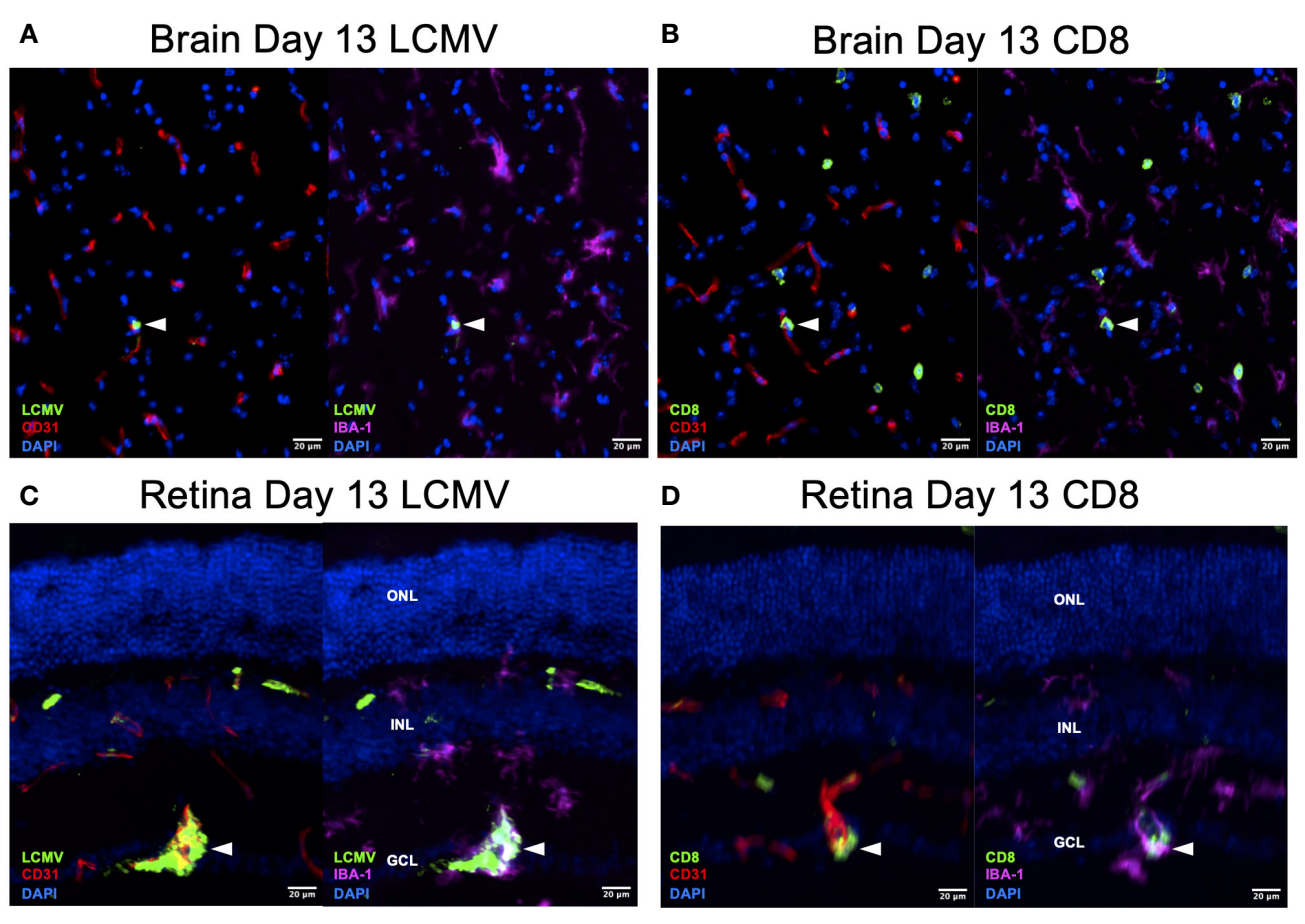
LCMV staining on Day 13 confirms active viral infection. **(A, B)** Immunofluorescence images of ×20 serial brain sections showing LMCV particles in close proximity to macrophages, endothelial cells, and CD8^+^ T cells (white arrowheads) on day 13. Scale bars are 20 µm. **(C, D)** Immunofluorescence images of ×20 serial retinal sections showing LMCV particles in close proximity to macrophages, endothelial cells, and CD8^+^ T cells (white arrowheads) on day 13. Scale bars are 20 µm. ONL, outer nuclear layer; INL, inner nuclear layer; GCL, ganglion cell layer.

To confirm that this T-cell difference is a response to infection rather than an immunologic phenomenon, we stained the day 13 retina and brain for LCMV. We found that LCMV localized to endothelial cells and macrophages in the brain and retina (**Figures 7A, C**). We also detected CD8^+^ T cells in similar regions on serial sections (**Figures 7B, D**). These data are correlative but could suggest that retinal microglia respond to LCMV infection and promote a greater CD8^+^ T-cell response compared to brain microglia.

## 3 Discussion

To determine the influence of the microenvironment on microglia function and gene expression, we compared microglia isolated from the brain and eye using scRNA-seq. After integration and identification of microglia, DE and GO analyses found that retinal microglia were enriched for antigen presentation by MHC class I and class II, cell chemotaxis, and T-cell differentiation and immunity compared to brain microglia (**Figure 4**). We validated these findings using multi-parameter flow cytometry, demonstrating that retinal microglia expressed more MHC class I receptors, H2-Db and H2-Kb, than brain microglia (**Figure 5**). Finally, we identified that the retina mounted a greater CD8^+^ T-cell response to chronic, neurotropic LCMV clone 13 infection than the brain. These results may explain differential responses between the retina and brain in viral infections and T-cell-mediated diseases.

Retinal microglia were found to exhibit a more inflammatory profile compared to their brain counterparts. This transcriptional profile includes greater antigen presentation, cell chemotaxis, and T-cell-mediated immunity. A potential explanation for this finding could be the unique anatomy of each organ. The retina lacks mechanical protection from the skull, exists within 200–300 µm of the highly fenestrated choroidal vasculature, and is devoid of lymphatics (22). These differences may increase retinal antigenic exposure compared to the brain, necessitating the need for a more rapid and robust inflammatory response. These results may explain why retinal autoimmune disease is more common than cerebral autoimmunity. For example, posterior uveitis has an estimated incidence of eight cases per 100,000 person-years (23), while multiple sclerosis, the most common cerebral autoimmune disease, has an incidence of 3.6 cases per 100,000 person-years (24).

We also demonstrated that retinal microglia express higher levels of MHC class I genes compared to brain microglia. Microglia are known to have the ability to cross-present antigens to CD8^+^ T cells via MHC class I (25), and this capability is instrumental in viral defense (26). Specifically, the MHC class I receptor H2-Db is necessary for both Theiler’s murine encephalomyelitis virus (27) and LCMV (28) presentation to CD8^+^ T cells. Furthermore, LCMV can directly infect macrophages, leading to antigen presentation on MHC class I receptors (28, 29). Therefore, our data demonstrating a greater CD8^+^ T-cell density in the retina compared to the brain corroborates not only MHC class I receptor expression in retinal microglia but also validates our findings of greater T-cell-mediated immunity in the retina. The greater incidence of herpes simplex virus retinitis (10–20 cases per million) compared to encephalitis (2.2–4.6 cases per million) exemplifies the importance of retinal viral immunity (30–32). We also found that ocular macrophages were enriched for MHC class 1 genes by scRNA-seq (**Supplementary Figure S2**). However, flow cytometry validated neither H2-Kb nor H2-Db in retinal macrophages (**Figure 5**). Therefore, our data support microglia as key MHC class 1 presenting cells in the retina, but do not rule out retinal hyalocytes or perivascular macrophages.

The balance between immunity and neurodegeneration may explain the differences between retinal and cerebral microglia. An excessive infiltration of microglial-activated T cells in the CNS can trigger tau-dependent neurodegeneration (33). Given the eye’s proximity to the external environment, this heightened antigen presentation might be a critical defense mechanism, while in the brain, minimizing neurodegeneration might be the priority, warranting a delicate balance. These data may explain the disconnect between AMD and AD regarding ApoE2/E4 risk. The ApoE4 allele is a risk factor for AD (13), but protective for AMD (14), while the ApoE2 allele is a risk factor for AMD and protective for AD (15, 16). ApoE is expressed by microglia and can induce Galectin-3 expression, resulting in neurodegeneration (34, 35). Thus, brain microglia may prioritize minimizing damage, inflammation, and neurodegeneration, while retinal microglia may prioritize antigen presentation and inflammation given their close proximity to the external environment.

Our study has several limitations. First, scRNA-seq is inherently lacking in sequencing depth. Second, because eyes are so small and despite using 20 eyes for our sample, we captured a smaller number of retinal microglia in our samples compared to brain microglia. However, the median genes per cell were nearly identical. The cell numbers and sequencing depths may affect our findings, but the validation of both MHC class I receptor expression by flow and its importance by systemic, persistent, neurotropic infection with LCMV was carried out (19, 20). Third, nonmicroglia macrophages from the eye include choroidal, iris, ciliary, and corneal macrophages, while nonmicroglial macrophages from the brain include border-associated macrophages and perivascular macrophages. These microenvironments and origins are very different, confounding these results. However, differences between nonmicroglial macrophages between the eye and brain are not a key result in this study and were only done from completeness. Finally, while mice and humans share similarities in their MHC class I molecular structure, they exhibit differences in gene organization and number (36). While our findings in mice provide valuable insights into the differential expression of MHC class I in the retinal and brain microglia, it is important to carefully consider how the expression of these genes can be extrapolated to human conditions.

In summary, we compared retinal and brain microglia using scRNA-seq data. We found greater MHC class I receptor expression and increased enrichment for T-cell-mediated immunity in the retina. We validated these findings by showing greater MHC class I receptor expression in retinal microglia compared to the brain. Furthermore, we demonstrated that LCMV infection caused greater T-cell-driven inflammation in the retina. These data demonstrate the importance of the microenvironment on microglia function and potentially answer intriguing questions regarding the differential responses of the retina and brain to infection and autoimmunity.

## 4 Methods

### 4.1 Mice

The original C57BL/6J breeders (No. 000664) were obtained from Jackson Labs (Bar Harbor, ME, USA) and bred in-house. *Tmem119^GFP/GFP^* mice (No. 031823) were obtained from Jackson Labs and were crossed with wild-type mice to generate *Tmem119^GFP/+^* mice for experiments. Animals were housed and bred in a barrier facility with unlimited access to food and water at the Center for Comparative Medicine at Northwestern University (Chicago, IL, USA). This study used 10- to 12-week-old mice. All procedures were approved by the Northwestern University Institutional Animal Care and Use Committee (IACUC).

### 4.2 Sex as a biological variable

All experiments were performed on female mice. This decision was made to reduce variability between sexes and because most immunologic diseases are more prevalent in females compared to males. It is unknown whether these findings are relevant in male mice.

### 4.3 Sample preparation for single-cell RNA-seq

Brains from three wild-type mice were harvested, dissected to remove meninges, and digested into a single-cell suspension as previously described (37). Single-cell suspensions were combined to yield a single biologically diverse sample. The single cell suspension was stained for viability dye, CD45, lineage markers (CD4, CD8, B220, NK1.1, Ly6G, SiglecF), and CD11b (**Table 1**). Fluorescence-activated cell sorting (FACS) was performed on a BD FACSAria cell sorter (BD Biosciences, Franklin Lakes, New Jersey, USA) at the Northwestern University RHLCCC Flow Cytometry Core Facility to isolate live, CD45^+^, CD11b^+^, Lineage^neg^ cells for scRNA-seq.

Eyes from 10 wild-type mice were collected and prepared as previously described (38). Briefly, the eyes were enucleated and dissected to remove conjunctiva, orbital fat, optic nerve, and extraocular muscles. A biologically diverse sample was obtained by combining 20 whole eyes, including cornea, iris, ciliary body, lens, vitreous, retina, choroid, retinal pigment epithelium, and sclera. Eyes were digested into a single-cell suspension, enriched for CD45^+^ cells (Miltenyi Biotec, 130-052-301), and stained for viability dye, CD45, and CD11b (**Table 1**) as previously described (39). FACS was performed on a BD FACS Aria cell sorter (BD Biosciences) at the Northwestern University RHLCCC Flow Cytometry Core Facility to isolate live CD45^+^ cells for scRNA-seq.

Brain and eye samples were sorted into MACS buffer (130-091-221, Miltenyi Biotec, Gaithersburg, Maryland, USA ), centrifuged at 350×*g* for 10 min at 4°C, and resuspended in RPMI (R8758, Millipore Sigma, Burlington, Massachusetts, USA). scRNA-seq libraries were prepared using the Single Cell 3’ v3.0 Reagent Kit and the 10× Genomics Chromium Controller in the Northwestern Metabolomics Core Facility. Sequencing was performed on the Illumina HiSeq 4000 platform. The Cell Ranger 3.1.0 (eye) and 6.0.0 (brain) pipeline (10× Genomics) were used for demultiplexing, trimming, alignment of reads to the genome, and exclusion of empty droplets. After filtering the scRNA-seq data, we detected 5,411 cells with 54,770 reads per cell (median genes per cell: 2,028) from wild-type eyes (GSE222094) and 12,543 cells with 23,746 reads per cell (median genes per cell: 1,917) from wild-type brains (GSE262152).

### 4.4 Bioinformatics

Data from brain and eye sample preparations were exported from the Cell Ranger pipeline as individual filtered_feature_bc_matrix output files and were analyzed using the Seurat v3 (40) package in the R Studio platform. Quality control analysis was done to remove cells with less than 200 features (empty droplets), more than 5,000 features (doublets), and greater than 25% mitochondrial DNA content (dead cells). Cells were then filtered again to remove low-quality cells with both greater than 8% mitochondrial DNA content and less than 1,000 features. Next, data were normalized (LogNormalize, scale.facor = 10,000), variable features were found (method = vst, 2,000 features), data were scaled using ScaleData, and RunPCA (npcs = 50) was performed on each object. An elbow plot was used to determine the number of principal components to include. Cells were clustered using FindNeighbors (dimensions = 1:12), FindClusters (resolution = 0.4), and visualized by RunUMAP. After clustering, cells were identified by canonical marker genes, including *Aif1*, *C1qa*, and *Fcgr1* for all macrophages. Next, *Siglech*, *Tmem119*, and *P2ry12* were used to identify microglia; *Ms4a7*, *Mrc1*, and *Cd163* identified nonmicroglia macrophages. The DotPlot function was used to visualize canonical markers from all identified cell types in each sample. The subset function was used to select all microglia and macrophages from the brain and eye independently.

Brain and eye macrophage subsets were integrated (FindIntegrationAnchors, IntegrateData) as previously described (41). The above process of normalization, finding variable features, scaling data, and performing RunPCA was repeated to then recluster the integrated macrophage subset (dimensions = 1:12, resolution = 0.4).

DE analysis using FindAllMarkers (min.pct = 0.25, fold change = 2) was performed on the integrated subset (**Supplementary Table S1**). We compared all brain macrophages to all eye macrophages, brain microglia to eye microglia, and brain border-associated macrophages to eye non-microglia macrophages. DE genes were visualized using the VolcanoPlot function. Hypergeometric distribution analysis was used to determine overlapping DE genes by tissue (*p* < 1.00 E−10). GO enrichment using Gorilla (42) was performed on brain and eye-upregulated genes (> 2-fold, adjusted *p*-value < 0.05), using a background of genes in > 25% of all macrophages (**Supplementary Table S2**).

DE analysis was also performed to assess for microglia heterogeneity. We used FindAllMarkers (min.pct = 0.25, fold change = 1.35) on the integrated subset of microglia comparing each microglia cluster versus all other microglia clusters (**Supplementary Table S3**). The DotPlot function was used to visualize the unique transcriptomic features of each microglia cluster. Hypergeometric enrichment analysis was performed using the phyper function to determine which tissue specifically contributed to each cluster (*p* < 0.001). GO enrichment using Gorilla and Enrichr pathway analysis (43, 44) was performed for each microglia cluster on upregulated genes (> 1.35-fold, adjusted *p*-value < 0.05), using a background of genes in > 25% of microglia (**Supplementary Table S4**).

### 4.5 Multiparameter flow cytometry analysis

Female *Tmem119^GFP/+^* (Jackson Labs No. 031823) mice aged 10- to 12-week-old were perfused with ice-cold 1× HBSS. Brains were harvested as previously described (45). Briefly, the tops of the skulls were removed, and brains were disconnected from the skull base by placing a spatula under the cerebellum and gently pushing it toward the olfactory bulbs. Brains were then scooped out by lifting and transferred to a Petri dish containing ice-cold sterile 1× HBSS. Using fine forceps, the meninges were freed near the olfactory bulbs and peeled back from the surface of the cortical layer while moving toward the cerebellum. To ensure thorough removal, brains were flipped to remove any remaining meninges from the basal side, with a careful inspection to confirm the absence of meningeal residue. The meninges-free brains were digested into a single-cell suspension as previously described (37). Eyes were dissected to isolate only the retina and digested as previously described (18). Cells were stained with live/dead Aqua viability dye (Invitrogen Waltham, Massachusetts, USA), incubated with Fc-Block (BD Biosciences), and stained with fluorochrome-conjugated antibodies (**Table 1**). Samples were run on a BD FACSSymphony A5-Laser Analyzer at the Northwestern University RHLCCC Flow Cytometry Core Facility. Cell types were identified based on canonical markers (**Supplementary Figures S1**, **S2**). The mean fluorescence intensity (MFI) of MHCI (H2-Db and H2-Kb) expression was determined on each defined cell population. Fluorescence-minus-one controls were used to define positive staining for H2-Db and H2-Kb. The final MFI was calculated by subtracting the FMO MFI from H2-Db and H2-Kb MFI for each population.

### 4.6 LCMV studies

Female 4–6-week-old wild-type C57BL6/J mice were infected with LCMV Clone 13 virus via retro-orbital injection (right eye) at 2 × 10^6^ plaque-forming units to create a persistent, chronic, and neurotropic LCMV infection (19, 20). Mice were euthanized on days 13 and 29 postinfection. Eyes were processed for retinal flat mounts and frozen sections (one eye each) identically to our previous study using 8-µm sections (18). Brains were removed from the skull and bisected through the median longitudinal fissure. Brains were fixed in 4% paraformaldehyde (No. 15713-S; Electron Microscopy Sciences, Hatfield, PA, USA) for 2 h at room temperature. Next, brains were washed in PBS with 10%, 20%, and 30% sucrose. Next, the brains were moved to a 25 mm × 20 mm × 5 mm vinyl specimen mold (4557, Sakura, CA, USA) with the sliced, medial side on the bottom of the mold, embedded in an optical cutting temperature compound (No. 23-730-571, Fisher Healthcare, Pittsburgh, PA, USA), and frozen at −80°C. A cryostat was used to make 12-µm sections. All sections were placed on HistoBond microscope slides (No. 16004-406, VWR; Batavia, IL, USA).

Eye and brain sections were stained identically. Briefly, slides were washed for 10 min in PBS and blocked at room temperature in PBS with 0.5% Tween-20 (PBS-T, No. 00777; Amresco, Solon, OH, USA) with 5% donkey serum (No. S30, Sigma-Aldrich, St. Louis, MO, USA) for 1 h. Primary incubations were performed at 4°C overnight (**Table 1**). Next, slides were washed with PBS-T five times and incubated with secondary antibodies for 1 h at room temperature (**Table 1**). Slides were then washed with PBS-T and mounted with Immu-Mount (No. 9990402; Thermo Fisher, Carlsbad, CA, USA). Immunofluorescence was performed on a Nikon Ti2 Widefield using Nikon NIS-Elements software.

Retinal flat mounts were stained identically, substituting Tris-buffered saline (TBS-T) for PBS-T. Blocking, primary, and secondary antibody incubations are performed overnight but otherwise identically to frozen sections. Imaging of the superficial and deep vascular plexuses was performed on a Nikon W1 Dual CAM Spinning Disk Microscope using Nikon NIS-Elements software.

T-cell densities were generated by manually quantifying the number of T cells in four ×20 fields. For retina flat mounts, we used four ×20 fields centered on the optic nerve in both the superficial and deep vascular plexuses. For brains, because T-cell density was heterogeneous but not region-specific (**Supplementary Figure S5**), we quantified four discreet ×20 fields that included a diverse array of T-cell densities from the midsagittal section. The four areas were then averaged.

### 4.7 Statistics

Hypergeometric enrichment analysis was performed with the phyper function in the R stats (v4.1.0) package. Enrichment was deemed significant at the *α* = 0.05 level with a Bonferroni correction for testing multiple hypotheses. MHCI Db and MHCI Kb percent and fold expression were compared using two-way ANOVA followed by Sidak’s multiple comparisons test. T-cell densities were compared using two-way ANOVA followed by Sidak’s multiple comparisons test. Student’s paired *t*-test was used to compare CD8^+^ T-cell density between superficial and deep plexuses and between retrobulbar and fellow eyes.

### 4.8 Study approval

All studies were performed in accordance with protocols approved by the Northwestern University Institutional Animal Care and Use Committee.

## Data availability statement

The datasets presented in this study can be found in online repositories. The names of the repository/repositories and accession number(s) can be found below: GSE222094 and GSE262152 (GEO).

## Ethics statement

The animal study was approved by Northwestern University Institutional Animal Care and Use Committee. The study was conducted in accordance with the local legislation and institutional requirements.

## Author contributions

CB: Writing – original draft, Visualization, Software, Investigation, Formal analysis, Data curation. JG: Writing – original draft, Visualization, Investigation, Formal analysis, Data curation. SD: Writing – review & editing, Investigation, Formal analysis, Data curation. HM: Writing – review & editing, Investigation. MG: Writing – review & editing, Investigation. CS: Writing – review & editing, Investigation. AK: Writing – review & editing, Investigation. GG: Writing – review and editing, Formal analysis. DW: Writing - review and editing, Resources. WC: Writing – review & editing, Resources. CC: Writing – review & editing, Supervision, Resources, Funding acquisition, Formal analysis, Data curation, Conceptualization. JL: Writing – original draft, Supervision, Resources, Funding acquisition, Formal analysis, Data curation, Conceptualization.
