# Supplementary material for: Retinal microglia express more MHC class I and promote greater T-cell-driven inflammation than brain microglia
Source: Front Immunol. 2024 May 10;15:1399989. doi: 10.3389/fimmu.2024.1399989 (PMC11116593; doi:10.3389/fimmu.2024.1399989)
Supplement: Supplementary file 1 [file DataSheet_1.docx]

Supplementary Material

# Supplementary Data

**File Name** Table S1

**File Format** Excel file

**Title of Data** Differential RNA expression between brain and eye

**Description of Data**: Global tab is all cells. Microglia tab is for microglia. Macrophage tab for non-microglia macrophages. Pct.1 = brain. Pct.2 = eye. Avg_log2FC = log2 fold change, pct.1 = percent of cells in brain that express the gene, pc.2 = percent of cells in retina that express the gene, p_val_adj = adjusted p-value for multiple comparisons.

**File Name** Table S2

**File Format** Excel file

**Title of Data** GO enrichment terms for global, microglia, and macrophage DE analysis.

**Description of Data**: Each tab denotes which tissue is upregulated (eye vs brain) and which cells (global, microglia, macrophage). Columns show GO term ID, description of GO term, p-value, FDR q-value, fold enrichment, number of genes expressed in macrophages (N), number of genes in GO term (B), number of differentially expressed genes (n), number of differentially expressed genes in the GO term (b), and the specific genes that were differentially expressed in the GO term. Any analysis not included had no significant GO enrichment.

**File Name** Table S3

**File Format** Excel file

**Title of Data** Differential RNA expression for each microglia cluster

**Description of Data**: Avg_log2FC = log2 fold change, pct.1 = percent of cells in cluster that express the gene, pct.2 = percent of all other cells that express the gene, p_val_adj = adjusted p-value for multiple comparisons.

**File Name** Table S4

**File Format** Excel file

**Title of Data** GO enrichment terms for microglia cluster DE analysis

**Description of Data**: Each tab denotes a microglia cluster. Any missing clusters had no significant GO term enrichment. Columns show GO term ID, description of GO term, p-value, FDR q-value, fold enrichment, number of genes expressed in macrophages (N), number of genes in GO term (B), number of differentially expressed genes (n), number of differentially expressed genes in the GO term (b), and the specific genes that were differentially expressed in the GO term.

# Supplementary Figures

**Figure S1. Canonical markers from microglia scRNA-seq**. Violin plots demonstrate similar expression levels of canonical markers Tmem119 and P2ry12 despite greater sequencing depth in eyes. Number of RNA features is slightly greater in retinal microglia.

**Figure S2. MHC Class 1 gene expression in microglia and macrophages**. Violin plots demonstrate that MHC class 1 genes are differentially expressed in ocular vs brain microglia (A) to a greater degree than ocular vs brain macrophages (B).

**Figure S3. Flow cytometry gating strategy for brain**. Multi-parameter flow cytometry gating strategy to identify terminal gates in red. Single, followed by CD45^+^, cells were identified. From CD45^+^ cells, dead cells were next removed. CD11b^+^Lin^neg^ cells were gated forward. From CD11b^+^Lin^neg^ cells, CD64^+^ macrophages were gated forward. From CD64^+^ macrophages, Cx3cr1^+^GFP^+^ cells were defined as microglia. GFP^neg^ cells were gated forward and CD45^high^ cells were identified as macrophages.

**Figure S4. Flow cytometry gating strategy for retina**. Multi-parameter flow cytometry gating strategy to identify terminal gates in red. Single, followed by live, cells were gated forward. Next, CD45^+^ cells were identified. From CD45^+^ cells, the gating strategy was identical to brain to delineate CD64^+^GFP^+^Cx3cr1^+^ microglia and CD64^+^GFP^neg^ macrophages.


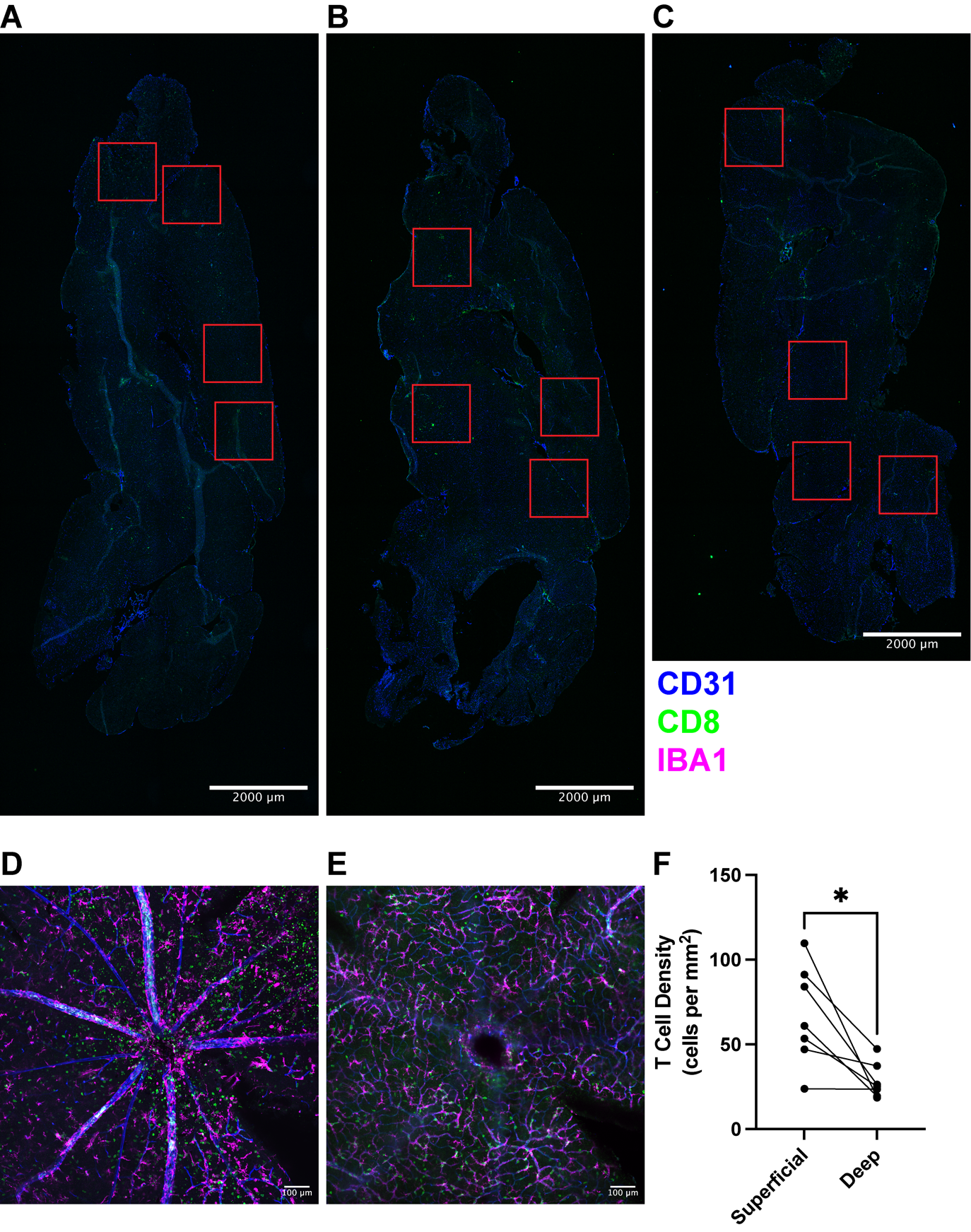



**Figure S5. LCMV imaging supplement.** **A-C**. Entire mid-sagittal sections from each brain at Day 13. Red boxes indicate the area used for quantification. **D-E**. Representative superficial and deep vascular plexus of the retina. **F**. The superficial vascular plexus showed greater T-cell density than the deep plexus on Day 13 and 29. * p<0.05. Student’s paired t-test (n=7 per group).

**Figure S6. T-cell density is not significantly different between eyes. A**. Representative retinal flatmount of the deep capillary plexus from the eye that received the retro-orbital injection. White arrowheads indicate CD8^+^ T-cells. **B**. Representative retinal flatmount of the deep capillary plexus from the fellow eye that did not receive the retro-orbital injection. **C**. Relative T-cell density between eyes showing no significant difference between eyes.
